# Investigation of genetically regulated gene expression and response to treatment in rheumatoid arthritis highlights an association between *IL18RAP* expression and treatment response

**DOI:** 10.1136/annrheumdis-2020-217204

**Published:** 2020-07-30

**Authors:** Svetlana Cherlin, Myles J Lewis, Darren Plant, Nisha Nair, Katriona Goldmann, Evan Tzanis, Michael R Barnes, Paul McKeigue, Jennifer H Barrett, Costantino Pitzalis, Anne Barton, MATURA Consortium, Heather J Cordell

**Affiliations:** 1 Population Health Sciences Institute, Faculty of Medical Sciences, Newcastle University, Newcastle upon Tyne, Tyne and Wear, UK; 2 Centre for Experimental Medicine and Rheumatology, William Harvey Research Institute, Barts and the London School of Medicine and Dentistry, Queen Mary University of London, London, UK; 3 Centre of Genetics & Genomics Versus Arthritis, Manchester Academic Health Science Centre, The University of Manchester, Manchester, UK; 4 NIHR Manchester Biomedical Research Centre, Manchester University NHS Foundation Trust, Manchester Academic Health Science Centre, Manchester, UK; 5 Centre for Population Health Sciences, Usher Institute of Population Health Sciences and Informatics, University of Edinburgh, Edinburgh, UK; 6 NIHR Leeds Biomedical Research Centre, Leeds Teaching Hospitals NHS Trust, Leeds, UK; 7 School of Medicine, University of Leeds, Leeds, UK

**Keywords:** rheumatoid arthritis, treatment, pharmacogenetics

## Abstract

**Objectives:**

In this study, we sought to investigate whether there was any association between genetically regulated gene expression (as predicted using various reference panels) and anti-tumour necrosis factor (anti-TNF) treatment response (change in erythrocyte sedimentation rate (ESR)) using 3158 European ancestry patients with rheumatoid arthritis.

**Methods:**

The genetically regulated portion of gene expression was estimated in the full cohort of 3158 subjects (as well as within a subcohort consisting of 1575 UK patients) using the PrediXcan software package with three different reference panels. Estimated expression was tested for association with anti-TNF treatment response. As a replication/validation experiment, we also investigated the correlation between change in ESR with measured gene expression at the *Interleukin 18 Receptor Accessory Protein* (*IL18RAP*) gene in whole blood and synovial tissue, using an independent replication data set of patients receiving conventional synthetic disease modifying anti-rheumatic drugs, with directly measured (via RNA sequencing) gene expression.

**Results:**

We found that predicted expression of *IL18RAP* showed a consistent signal of association with treatment response across the reference panels. In our independent replication data set, *IL18RAP* expression in whole blood showed correlation with the change in ESR between baseline and follow-up (*r*=*−*0.35, p=0.0091). Change in ESR was also correlated with the expression of *IL18RAP* in synovial tissue (*r*=*−*0.28, p=0.02).

**Conclusion:**

Our results suggest that *IL18RAP* expression is worthy of further investigation as a potential predictor of treatment response in rheumatoid arthritis that is not specific to a particular drug type.

**Key messages**

**What is already known about this subject?**

- IL-18 plays an inflammatory role in rheumatoid arthritis and has previously been identified as a potential therapeutic target.
- The protein encoded by the gene *IL18RAP* enhances the IL-18-binding activity of the IL-18 receptor and plays a role in IL-18 signalling.

**What does this study add?**

- We demonstrate a robust association between *IL18RAP* gene expression (both in whole blood and in synovial tissue) and treatment response in rheumatoid arthritis.
- The association between *IL18RAP* expression and treatment response is not specific to a particular drug type but is observed across different treatments.

**How might this impact on clinical practice or future developments?**

- Measurements of *IL18RAP* expression could potentially be incorporated into a multiomic predictive model for treatment response in rheumatoid arthritis in the future.

## Introduction

Tumour necrosis factor (TNF) α inhibitors (anti-TNFs) are the most commonly prescribed second-line drugs for patients with conventional synthetic disease modifying anti-rheumatic drug (csDMARD)-resistant rheumatoid arthritis (RA). However, patients show a significant non-response rate to anti-TNF treatment.^1 2^ With recent advances in microarray and RNA sequencing (RNA-seq) technologies, it is hypothesised that gene expression profiling might inform our understanding of the heterogeneity of responses to treatment in RA.^3^ Indeed, Tanino *et al* 4 identified 10 genes predictive of response to the anti-TNF antibody infliximab, based on a transcriptome analysis of white blood cells from patients with RA, while Julià *et al* 5 identified an eight-gene predictor model from microarray gene expression analysis on whole blood RNA samples from patients with RA. Previously, using a microarray analysis of mononuclear cell RNAs, Lequerré *et al* 6 was able to perfectly separate responders to infliximab from non-responders.

However, when studying a large number of patients, measuring gene expression at a genome-wide scale might not be financially feasible, as RNA-seq remains more expensive than genome-wide genotyping approaches. The PrediXcan method/software package^7^ is a cost-effective approach for estimating the genetically regulated portion of gene expression at each gene from a genome-wide set of genes. PrediXcan estimates the component of a gene’s expression determined by an individual’s single nucleotide polymorphism (SNP) genotypes (at SNPs in the vicinity of the gene) and then tests for association between the predicted expression and the phenotype. The estimation of gene expression is performed using whole-genome tissue-dependent prediction models trained with reference panels that have both SNP and gene expression data. Here, we applied PrediXcan to data on patients with RA receiving anti-TNF treatment from the MAximising Therapeutic Utility for Rheumatoid Arthritis (MATURA) consortium,^8^ focusing on testing the association between the change in erythrocyte sedimentation rate (ESR) and predicted gene expression. We elected to focus on change in ESR as an objective measure of response that has been shown to have higher heritability than other measures of response.^9^

## Methods

### UK data set

The UK data set was comprised of imputed genome-wide SNP genotype data (9 084 265 SNPs) for up to 1583 patients receiving anti-TNF treatment from the MATURA consortium; this corresponds to the ‘anti-TNF, ESR data set’ previously described by Cherlin *et al*.^10^ Quality control (QC) on the imputed SNP data was performed using standard procedures outlined by Anderson *et al*.^11^ Individuals were excluded if the reported sex did not match the sex assessed by genotype, and samples with elevated missingness rate, outlying heterozygosity rate, outlying ethnicity and relatedness were also excluded. SNPs were excluded if they had a postimputation INFO score *<*0.8. Genotype hard calls were set to missing if the posterior probability was *<*0.9. The data was filtered by minor allele frequency (*>*0.01), Hardy–Weinberg disequilibrium (p>0.000001) and missing genotype rate (*<*0.05). The SNP genotypes were encoded according to the number of copies of the minor allele possessed. The phenotype was defined as the difference between the follow-up ESR measure (measured at 6 months, or 3 months if this was not available) and the baseline ESR measure on the log scale, that is, log(ESR*fu*) *−* log(ESR*bl*). This difference was then adjusted (by taking as the final phenotype the standardised residuals from a linear regression, carried out in the statistical software package R) for baseline ESR, drug type (a five-level categorical variable indicating adalimumab, etanercept, infliximab, certolizumab pegol and golimumab), a separate binary indication of whether or not patients received another DMARD in addition to the anti-TNF treatment, gender and the first 10 principal components (PCs) of the SNP genotypes. The final post-QC data set was comprised of 1575 individuals and 4 542 023 SNPs.

### Expanded European ancestry data set

An expanded European ancestry data set was constructed, consisting of imputed genotype data at 4 498 586 genome-wide SNPs for 3158 patients. This expanded data set consisted of a combination of the original (1575 patient) UK data set and a separate independent data set of 1583 US and EU patients, corresponding to a subset (to which we were granted access) of the patients from a pre-existing international collaboration formed to study the genetics of response to TNF inhibitors.^12^ The same QC procedures were performed separately on the 1583 US/EU patients, and their post-QC SNP genotype data were merged with the data for the 1575 UK patients. In this combined European ancestry data set, the phenotype, defined as the difference between the follow-up ESR measure and the baseline ESR measure on the log scale, was adjusted for the baseline ESR measure on the log scale (log(ESR*bl*)) and the first three PCs (which was found sufficient to produce no inflation in the genome-wide set of test statistics for association between SNPs and phenotype) and subsequently adjusted for gender; other covariates were not available. The standardised residuals after all adjustments were then taken as the final phenotype.

### Replication data set

The replication data set consisted of 90 treatment-naive early RA patients fulfilling the 2010 ACR/EULAR RA Classification Criteria from the Pathobiology of Early Arthritis Cohort (PEAC), in whom ultrasound-guided synovial biopsies (n=87 post-QC) and whole blood samples (n=67) were subject to RNA sequencing as previously described.^13^ Notably, both synovial biopsies and blood samples were taken prior to patients receiving any disease modifying treatment such as corticosteroids. One microgram of total RNA was used as an input material for library preparation using TruSeq RNA Sample Preparation Kit v2 (Illumina). Generated libraries were amplified with 10 cycles of PCR. Size of the libraries was confirmed using 2200 TapeStation and High Sensitivity D1K screen tape (Agilent Technologies) and concentration was determined by qPCR-based method using Library quantification kit (KAPA). Multiplexed libraries (five per lane) were sequenced on Illumina HiSeq2500 to generate 50 million paired-end 75 base pair reads. Transcript abundance was derived using Kallisto V.0.43.0 and tximport 1.4.0 using GENCODE V.24/GRCh38 as reference and transformed to regularised log expression (RLE) using DESeq2 1.14.1. RNA-seq data have been deposited in ArrayExpress under Accession code E-MTAB-6141. Genotyping and QC were performed using the same methodology as for the UK data set as described above. Expression quantitative trait locus (eQTL) analysis on PEAC blood RNA-seq samples was performed using the matrix eQTL package in R^14^ using four PCs to adjust for ancestry and four probabilistic estimation of expression residuals calculated as per Stegle *et al* 15 as model covariates.

### Discovery analysis based on predicted gene expression

PrediXcan was applied to both the UK data set and the expanded European ancestry data set. In PrediXcan, an elastic net prediction model is built using a reference panel containing SNP and gene expression data. This model is then used to predict expression levels in the analysis cohort on the basis of the measured genotypes, and the resulting predicted expression levels are tested for association with the outcome of interest. We applied PrediXcan using three reference panels: (1) a MATURA reference panel comprising 210 MATURA samples (a subset of the UK samples used later for testing), for which SNP and gene expression data were available)^8^; (2) a GTEx reference panel for which PrediXcan provides precalculated models; this panel comprises 338 samples from the Genotype-Tissue Expression Consortium;^16^ (3) a DGN reference panel for which PrediXcan provides precalculated models; this panel comprises 922 samples from the Depression Genes and Networks Consortium.^17^

To construct the MATURA reference panel, we used 33 170 QC-ed and batch-adjusted^18^ gene expression probes from the Illumina HT-12 Gene Expression Beadchip, measured in whole blood at baseline in 210 patients, together with QC-ed imputed genotype data at 3 978 972 genome-wide SNPs in the same patients. Probes that corresponded to different genes (according to the GENCODE V.19)^19^ were removed, and probes that corresponded to the same gene were combined as specified.^20^ The final gene expression data set consisted of 17 008 probes. We note that the SNPs used for constructing the MATURA reference panel corresponded to a subset of the SNPs present within the UK data set; however, they did not correspond to an exact subset of SNPs present in the expanded European ancestry data set owing to different SNPs remaining post-QC. We used default PrediXcan parameters to build the elastic net model (*α*=0.4, window size=1 Mbp, false discovery rate threshold=0.05). The final MATURA reference panel included 1573 genes. Models based on whole blood GTEx and DGN reference panels were provided as part of the PrediXcan software (downloaded from http://predictdb.org/) and included 6057 and 9836 genes, respectively.

### Replication analysis based on measured gene expression

Clinical parameters including DAS28 score and subcomponents, ESR, C-reactive protein (CRP), rheumatoid factor (RF) and anti-citrullinated peptide antibody positivity/titre were collected at baseline and every 3 months. Patients were treated with methotrexate-based combination DMARDs (81%), methotrexate alone (6.8%), hydroxychloroquine alone (5.9%) or sulfasalazine alone (3.4%) or no DMARDs (2.5%). Clinical response was assessed by change in clinical parameters at 6 months and correlated with *IL18RAP* transcript levels measured by RNA-seq in baseline synovial biopsy or whole blood.

## Results

### Discovery analysis based on predicted gene expression

**Figure 1:**
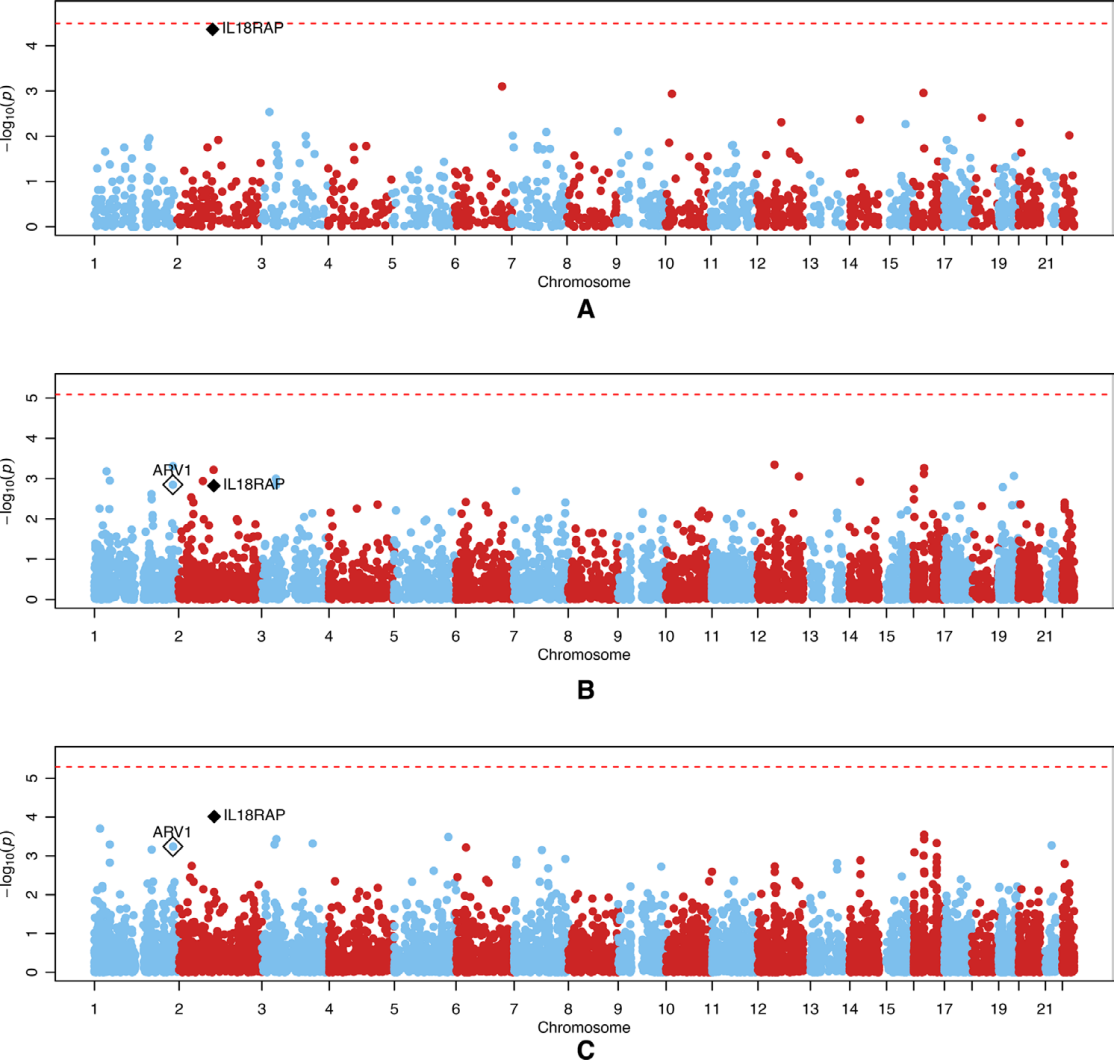
Manhattan plots of p values from tests of association between genetically regulated gene expression and the change in erythrocyte sedimentation rate for the 1575-person UK data set. The genetically regulated gene expression was estimated with (A) the MATURA reference panel, (B) the GTEx reference panel and (C) the DGN reference panel. On each panel, the red dashed line represents the experiment-wide significance level computed using a Bonferroni correction for the number of tests performed. The black diamond represents the *IL18RAP* gene. The white diamond represents the *ARV1* gene.

**Figure 2:**
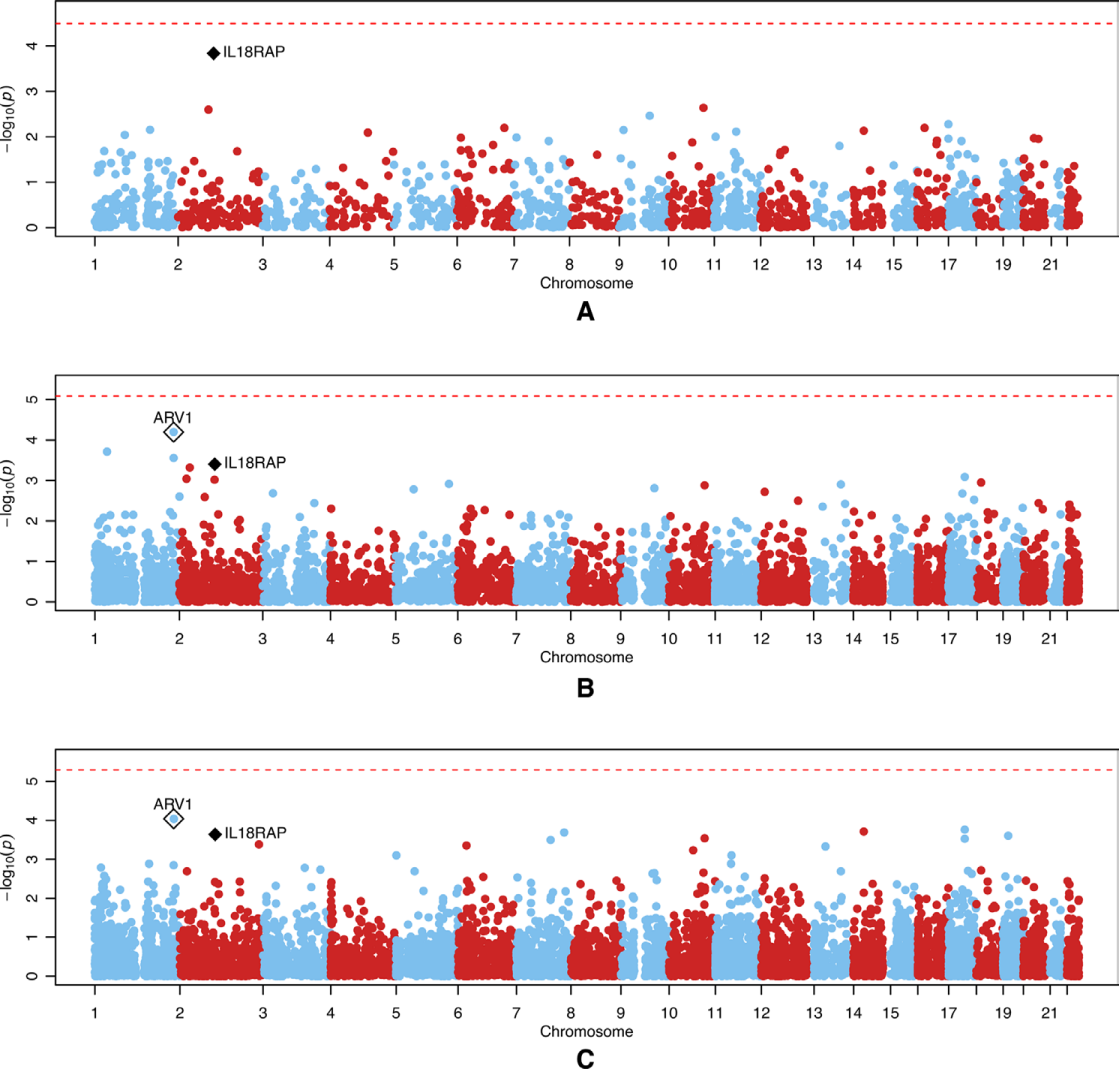
Manhattan plots of p values from tests of association between genetically regulated gene expression and the change in erythrocyte sedimentation rate for the 3158-person expanded European ancestry data set. The genetically regulated gene expression was estimated with (A) the MATURA reference panel, (B) the GTEx reference panel and (C) the DGN reference panel. On each panel, the red dashed line represents the experiment-wide significance level computed using a Bonferroni correction for the number of tests performed. The black diamond represents the *IL18RAP* gene. The white diamond represents the *ARV1* gene.

The results for the UK and expanded European ancestry data sets are shown in figures 1 and 2, respectively. Although no genes pass experiment-wide significance, for the UK data set (figure 1), the most significant gene identified using the MATURA reference panel (*IL18RAP* on chromosome 2) achieved close to experiment-wide significance (p=4.3*×*10^–5^), and this was also the top gene when using the DGN reference panel (p=6.7*×*10^–5^). When using the GTEx reference panel, *IL18RAP* was the fourth top gene (p=1.5*×*10^–3^).

For the expanded European ancestry data set (figure 2), *IL18RAP* was again the top gene when using the MATURA reference panel (p=1.4*×*10^–4^). With the DGN reference panel, this gene was the fifth top gene (p=2.3*×*10^–4^), and with the GTEx reference panel, it was the fourth top gene (p=4.0*×*10^–4^). Online supplementary table S1 shows the significance levels and effect estimates achieved at *IL18RAP* when using either the full 3158-person expanded European ancestry cohort or when it is divided into its constituent UK or US/EU subcohorts. (Note that the results for the UK subcohort differ slightly from those obtained in the original analysis of the UK data set on account of (a) the different phenotypic adjustments made when using the full expanded European ancestry data set and (b) slightly different SNPs being available for prediction of expression, see below). The signal is seen to be predominantly driven by the results from the UK subcohort, with the US/EU subcohort showing the same direction of effect, but with the effect size considerably attenuated.

10.1136/annrheumdis-2020-217204.supp1 Supplementary data

The PrediXcan models for predicting the expression of *IL18RAP* involved 77, 46 and 86 SNPs when using the MATURA, GTEx and DGN reference panels, respectively (online supplementary table S2). All 77 SNPs from the MATURA reference panel appeared in the expanded European ancestry data set, while 37 out of 46 SNPs from the GTEx reference panel and 82 out of 86 SNPs from the DGN reference panel appeared. Density estimates for the resulting predicted expression values are shown in online supplementary figure S1; their relationship with phenotype is shown in online supplementary figure S2. The prediction *R* ^2^ statistic for *IL18RAP* (based on PrediXcan’s internal 10-fold cross-validation procedure using the relevant reference panel) was 0.32 (p=1.9*×*10^–19^) with the MATURA reference panel, 0.30 (p=4.4*×*10^–247^) with the GTEx reference panel and 0.71 (p=2.8*×*10^–28^) with the DGN reference panel, suggesting reasonable predictive ability for expression at this gene across all panels. As expected (given the association between SNPs contributing to the prediction models and expression, and between predicted expression and response), a number of SNPs also showed direct associations with response (online supplementary table S2), although as noted previously^10^ these do not meet genome-wide significance levels.

The most significant gene overall using the expanded European ancestry data set was *ARV1* on chromosome 1, which appeared when using both the DGN reference panel (p=9.1*×*10^–5^) and the GTEx reference panel (p=6.4*×*10^–5^). This gene was absent on the MATURA reference panel because the PrediXcan software failed to predict its expression value. In the original analysis of the UK data set, the signals for the *ARV1* gene were generally weaker than, or similar to, those seen at *IL18RAP* (figure 1). Additionally, the prediction accuracy for *ARV1* in the expanded European ancestry data set, as measured by the *R* ^2^ statistic, was very low (*R* ^2^=9.6*×*10^–3^; p=0.072) with the GTEx reference panel and relatively lower (*R* ^2^=0.18; p=2.1*×*10^–41^) than that seen for *IL18RAP* with the DGN reference panel, suggesting that these results at *ARV1* should be interpreted with caution.

### Replication analysis based on measured gene expression

**Figure 3:**
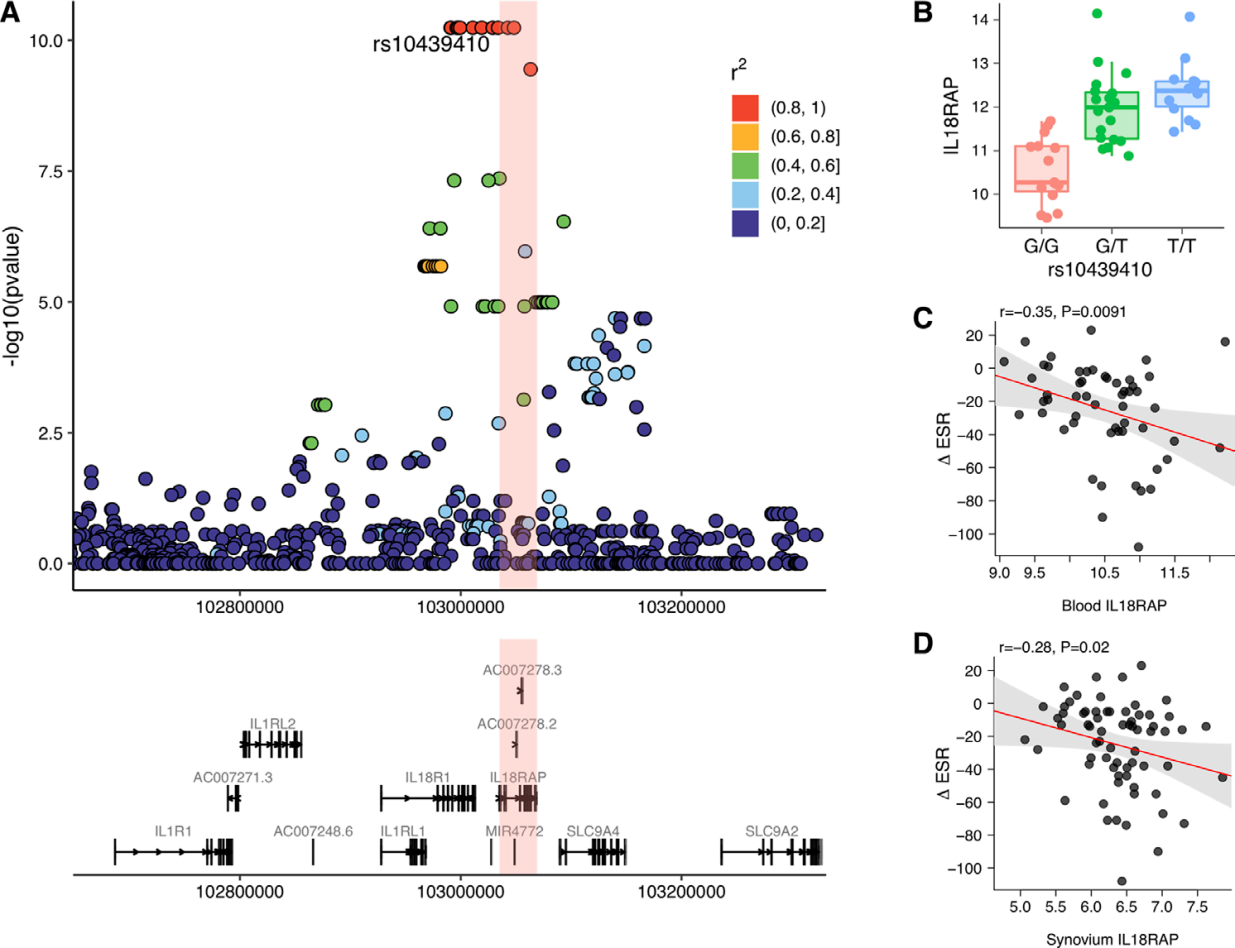
Confirmation of the *IL18RAP* expression quantitative trait locus and clinical consequences in rheumatoid arthritis. (A) Manhattan plot showing expression quantitative trait locus analysis comparing influence of SNPs at the *IL18RAP* locus on *IL18RAP* expression in blood measured by RNA-seq. (B) Scatter plot of SNP rs10439410 in the 5’ upstream region of *IL18RAP* and *IL18RAP* expression in whole blood. (C and D) Correlation between the change in erythrocyte sedimentation rate between baseline and 6 months of combination disease modifying anti-rheumatic drug therapy and *IL18RAP* expression measured by RNA-seq in whole blood (C) and synovial tissue (D).

In the replication data set, we observed a significant eQTL association (p=5.8*×*10^–11^) between multiple SNPs across the *IL18RAP* locus and *IL18RAP* expression measured by RNA-seq of whole blood samples in patients with early RA (figure 3A, B), thus confirming that *IL18RAP* genetic polymorphisms regulate expression of *IL18RAP* in peripheral blood in patients with early RA. The expression of *IL18RAP* measured in whole blood showed correlation with the change in ESR between baseline and 6-month follow-up (*r*=*−*0.35; p=0.0091) in patients with RA treated with methotrexate-based combination DMARD therapy (figure 3C); specifically each unit increase in *IL18RAP* RLE resulted in a 13.4 mm/hour decrease in ESR between baseline and 6 months. Also, a correlation was observed between the expression of *IL18RAP* in synovial tissue and the change in ESR (*r*=*−*0.28; p=0.02) (figure 3D); specifically, each unit increase in RLE resulted in a 11.8 mm/hour decrease in change in ESR over 6 months. Thus, our replication experiment based on actual measured gene expression (in an independent set of patients) validates the association between predicted *IL18RAP* expression and treatment response seen in the discovery cohort.

## Discussion

In this study, we investigated the association between the genetically regulated portion of gene expression and change in the ESR in a large cohort of patients with RA from the MATURA consortium. We found that predicted expression of *IL18RAP* showed a consistent signal across data sets analysed using different reference panels, while achieving a reasonable level of prediction accuracy as measured by the prediction *R* ^2^. Despite the consistency of the results for *IL18RAP*, some differences in the strength of the signal were observed for different data sets and reference panels. These differences require further investigation; however, they can be partly explained by the different sample sizes (and SNPs available to inform prediction) in the different reference panels. In an independent replication data set of patients treated with csDMARDs with measured gene expression, the association between expression of *IL18RAP* and change in the ESR was confirmed in both whole blood and synovial tissue, highlighting *IL18RAP* as a gene worthy of further investigation for prediction of treatment response in RA that is not treatment-specific. No other expressed genes were consistently associated with response, providing confidence that it is the *IL18RAP* gene that is driving the association rather than serving as a proxy for another gene.

The protein encoded by *IL18RAP* enhances the IL-18-binding activity of the IL-18 receptor and plays a role in signalling by IL-18.^21^ IL-18 plays an inflammatory role in RA^22 23^ and has previously been identified as a potential therapeutic target in the treatment of RA.^24 25^ It has been suggested that IL-18 plays some part in the degradation of articular cartilage in arthritis.^26^ Additionally, Rooney *et al* 27 showed that synovial tissue IL-18 production measured by immunohistochemistry was correlated with serum CRP in inflammatory arthritis, while Joosten *et al* 28 found a correlation between the level of IL-18 in the synovial tissue of the patients with RA and ESR.

Previous studies have reported a potential association between *IL18RAP* and treatment response in RA. Analysis of cap analysis of gene expression (CAGE) sequencing data from the FANTOM5 consortium showed that *IL18RAP* is highly expressed in neutrophils, gamma delta T cells, eosinophils and natural killer (NK) cells.^29^ Analysis of the BioGPS database (http://biogps.org/) confirms that *IL18RAP* is highly expressed in NK cells. *IL18RAP* expression is upregulated in NK and T cells in response to IFN-alpha and IL-12.^30^ *IL18RAP* was found to be significantly upregulated (adjusted p=5.5 *×* 10^–78^) in NK cells in single-cell RNA-seq RA synovium data from Stephenson *et al*.^31^ Similarly, in a second single-cell RNA-seq study of RA synovium,^32^ *IL18RAP* shows increased expression in synovial tissue T cell populations. In RA synovium, the baseline expression of the S1 module (NK cell surface signature) from Li *et al,* 33 which includes *IL18RAP* as one of its 45 genes, correlates significantly with change in ESR. Additionally, the synovial baseline expression for another NK cell module (M7.2), which includes *IL18RAP,* is also significant for the change in ESR.

Our own investigation of the relationship between measured expression of *IL18RAP* and change in the ESR in whole blood and synovial tissue in our replication data set was motivated by our initial identification of a relationship between change in the ESR and predicted expression of *IL18RAP* in our discovery data sets, using the PrediXcan method/software. Other methods/software packages for performing transcriptome-wide association studies exist, but as shown by Fryett *et al*,^34^ they tend to perform very similarly to one another. These methods are dependent on the underlying eQTL data used to build the prediction models, and therefore would generally be expected to give very similar results. Given that the external data sets used to inform the prediction were derived from population studies and would unlikely have been enriched for patients with RA, the risk of the association detected with ESR being spurious is low.

We elected to focus on change in ESR as an objective measure of response that has been shown to have higher heritability than other measures of response.^9^ Other clinical outcomes relating to anti-TNF treatment response, such as joint destruction scores or CRP, could certainly be assessed using similar approaches. However, joint destruction scores were not available in our discovery data set, and there were many missing values for CRP, making this a less attractive option in this instance.

Overall, our results, combined with the existing evidence, suggest that the expression of *IL18RAP* in whole blood might have utility for predicting response to treatment in RA. However, the effect observed in our replication samples (11.8–13.4 mm/hour decrease in change in ESR over 6 months) is, by itself, probably too small to be clinically useful, and the small to moderate correlations seen between *IL18RAP* expression and change in ESR (figure 3C, D) suggest that the actual predictive ability of *IL18RAP* expression alone may be limited. This approach shows the value of integrating genetic and expression data to identify factors correlated with response, which could be incorporated into a multiomic predictive model in the future. Further investigation of the relationship between *IL18RAP* expression and varying measures of treatment response in additional patient cohorts is thus warranted.
