# Supplementary material for: Investigation of genetically regulated gene expression and response to treatment in rheumatoid arthritis highlights an association between IL18RAP expression and treatment response
Source: Ann Rheum Dis. 2020 Jul 30;79(11):1446–52. doi: 10.1136/annrheumdis-2020-217204 (PMC7569378; doi:10.1136/annrheumdis-2020-217204)
Supplement: Supplementary data [file annrheumdis-2020-217204supp001.pdf]

Investigation of genetically-regulated gene expression and response to treatment in rheumatoid arthritis highlights an association between *IL18RAP* expression and treatment response

Online Supplementary Material

Table S1. Effect sizes and significance levels achieved at *IL18RAP* when using either the full 3158-person expanded European ancestry cohort or when it is divided into its constituent UK (1575 people) and US/EU (1583 people) sub-cohorts.

Reference panel	Gene	UK		US/EU		Full	
		Effect size	*P*-value	Effect size	*P*-value	Effect size	*P*-value
MATURA	*IL18RAP*	-0.210	0.00021	-0.054	0.17344	-0.131	0.00014
GTEx	*IL18RAP*	-0.235	0.00105	-0.076	0.11999	-0.152	0.00040
DGN	*IL18RAP*	-0.128	0.00025	-0.030	0.21817	-0.079	0.00023

Table S2. The columns "Effect MATURA", "Effect GTEx" and "Effect DGN" represent the effects of the SNPs in the prediction models when using the MATURA (77 SNPs), GTEx (46 SNPs) and DGN (86 SNPs) reference panels. The columns "Coeff" and "P-value" represent the coefficient and the P-value from the association between the SNPs and the phenotype in the UK data set.

SNP	BP	Effect MATURA	Effect GTEx	Effect DGN	Coeff	P-value
rs13413645	-	-	-0.040	-	-	-
rs6759556	-	-	0.216	-	-	-
rs2310220	-	-	0.094	-	-	-
rs11465677	-	-	-0.003	-	-	-
rs17775170	-	-	0.009	-	-	-
rs10178585	-	-	-0.028	-	-	-
rs10185170	-	-	0.068	-	-	-
rs12328682	-	-	0.004	-	-	-
rs7599071	-	-	-0.010	-	-	-
rs1093515	-	-	-	0.015	-	-
rs13427957	-	-	-	-0.015	-	-
rs266064	-	-	-	-0.004	-	-
rs4319952	-	-	-	-0.021	-	-
rs7589943	102038936	-	0.055	-	0.006	0.96600
rs290772	102126220	-	-	0.007	-0.065	0.34800
rs75094400	102142042	0.080	-	-	-0.236	0.02390
rs17201799	102179247	-	0.054	-	-0.038	0.37600
rs12990046	102543479	0.039	-	-	-0.007	0.85600
rs11678842	102674806	-0.010	-	-	0.074	0.08400
rs13388182	102722402	-	-	-0.020	0.032	0.38700
rs13029804	102723017	-	-	0.014	-0.072	0.11000
rs1812326	102860411	-0.031	-	-	0.069	0.05930
rs10186746	102866377	-0.026	-	-	0.065	0.07800
rs62151694	102899464	0.049	-	-	-0.072	0.25500
rs13007174	102960487	0.006	-	-	-0.081	0.17300
rs13014044	102961366	0.005	-	-	-0.087	0.14800
rs1946131	102961929	0.005	-	-	-0.077	0.19600
rs1054096	102962350	0.005	-	-	-0.079	0.18600
rs12989197	102962739	0.004	-	-	-0.075	0.21100
rs12996097	102963628	0.004	-	-	-0.081	0.17400
rs13028993	102963949	0.004	-	-	-0.076	0.20100
rs12999542	102965392	-	-	0.017	-0.083	0.16300
rs13014644	102971363	0.015	-	-	-0.079	0.18500
rs13015714	102971865	-	0.018	-	0.111	0.00955
rs11465567	102978400	0.006	-	-	-0.072	0.22800

rs11465572	102980223	0.001	-	-	-0.071	0.23200
rs4134504	102982223	0.005	-	-	-0.081	0.17000
rs2058622	102985424	-	-	0.016	0.112	0.00945
rs2058623	102986170	-	-	0.015	0.113	0.00863
rs1465321	102986618	-	-	0.002	0.113	0.00863
rs11465597	102987213	-	-	0.006	-0.057	0.33600
rs10439410	102990788	0.007	-	0.031	-0.143	0.00005
rs6731157	102991191	0.006	-	-	-0.143	0.00005
rs6745614	102991213	0.007	-	-	-0.143	0.00005
rs6758936	102991369	0.007	-	0.016	-0.143	0.00005
rs2270297	102992675	-	0.011	0.050	0.115	0.00729
rs11465623	102993039	-	0.058	0.019	-0.020	0.76900
rs6753717	102993161	-	0.012	0.048	0.115	0.00729
rs2041739	102994333	0.012	-	0.018	-0.143	0.00006
rs6750020	102994714	-	0.013	0.046	0.114	0.00807
rs10208196	102996345	0.007	-	0.019	-0.143	0.00005
rs7556917	102997720	0.008	-	-	-0.143	0.00005
rs7584093	102997721	0.008	-	-	-0.143	0.00006
rs3213733	102997884	-	-	0.001	-0.102	0.03120
rs3213732	102998279	0.008	-	0.021	-0.144	0.00005
rs10204757	102998974	0.008	-	-	-0.143	0.00005
rs6760621	102999952	0.008	-	0.021	-0.143	0.00005
rs11465641	103000868	0.009	-	-	-0.143	0.00005
rs17651485	103001650	-	0.057	0.086	-0.027	0.68000
rs3771161	103003961	-	-	0.001	-0.099	0.03470
rs3771159	103004958	0.009	-	-	-0.143	0.00005
rs11903946	103005330	0.009	-	-	-0.143	0.00005
rs6706002	103006104	0.009	-	0.023	-0.143	0.00005
rs6749014	103006448	0.009	-	0.022	-0.144	0.00005
rs12712146	103008714	0.006	-	-	-0.156	0.00002
rs4851004	103009537	0.009	-	0.023	-0.144	0.00005
rs6732138	103009662	0.009	-	-	-0.146	0.00004
rs3771158	103009894	-	-	0.018	-0.095	0.04320
rs1420096	103010912	0.009	-	-	-0.145	0.00005
rs2287033	103011237	0.009	-	0.022	-0.145	0.00005
rs1568681	103014696	-0.044	-	-	0.118	0.00606
rs1420094	103015687	0.011	-	0.023	-0.143	0.00005
rs6710528	103016142	0.009	-	0.022	-0.142	0.00006
rs3732124	103018052	-	-	0.022	-0.143	0.00005
rs4851571	103019000	-	-	0.022	-0.144	0.00005
rs4851572	103019031	0.009	-	0.022	-0.144	0.00005
rs10202813	103019740	-	-	0.014	-0.099	0.03460

rs1035129	103019785	0.009	-	-	-0.145	0.00005
rs1035127	103019919	-0.045	0.027	0.049	0.114	0.00767
rs6710034	103023678	0.010	-	0.021	-0.144	0.00005
rs7589142	103024660	0.010	-	-	-0.145	0.00004
rs4851007	103024813	-0.045	0.026	0.047	0.114	0.00767
rs4851575	103025203	-0.045	0.171	0.046	0.113	0.00811
rs10181785	103025274	-	-	0.010	-0.097	0.03900
rs12712148	103025547	-	-	0.007	-0.096	0.03990
rs11687768	103025738	-	-	0.005	-0.096	0.04110
rs6543126	103027104	0.009	-	-	-0.144	0.00005
rs10203558	103027640	0.007	-	0.021	-0.144	0.00005
rs10200952	103027651	0.007	-	0.021	-0.143	0.00006
rs7586983	103028066	-	-	0.008	-0.096	0.04110
rs6543127	103028301	0.009	-	-	-0.143	0.00005
rs4851576	103028895	0.012	-	0.022	-0.143	0.00005
rs4851577	103028921	0.017	-	0.021	-0.143	0.00005
rs4851579	103028984	0.009	-	0.021	-0.143	0.00005
rs6543132	103029410	-0.044	-	-	0.114	0.00799
rs1807782	103033147	-0.017	0.023	0.045	0.115	0.00765
rs1035125	103033839	0.005	-	-	-0.143	0.00005
rs2160201	103033961	0.017	-	-	-0.143	0.00005
rs1420106	103035044	-0.043	0.022	0.045	0.113	0.00857
rs1420105	103035119	0.017	-	0.020	-0.143	0.00005
rs11465673	103035375	-	-	0.010	-0.028	0.67400
rs2293223	103035468	-	-	0.003	-0.097	0.03880
rs2293224	103035779	0.009	-	0.019	-0.143	0.00005
rs6756407	103036101	-	-	0.011	-0.026	0.68800
rs6743516	103036335	0.009	-	0.019	-0.143	0.00005
rs1420100	103037002	0.008	-	0.019	-0.143	0.00005
rs3771155	103037826	0.009	-	0.021	-0.144	0.00005
rs3755267	103038587	-0.043	0.023	0.032	0.113	0.00860
rs10206291	103038863	0.009	-	0.030	-0.144	0.00005
rs885088	103039044	0.017	-	0.019	-0.143	0.00005
rs3771154	103039360	0.017	-	0.020	-0.143	0.00005
rs2272128	103039929	-0.042	0.023	0.029	0.116	0.00728
rs6759479	103040047	0.016	-	0.020	-0.143	0.00005
rs11465689	103040167	-	-	0.036	-0.030	0.65000
rs12997015	103042401	0.002	-	-	0.011	0.91800
rs3755266	103042712	-	-	0.021	-0.143	0.00005
rs11694658	103045020	-	-	0.006	0.108	0.01160
rs7559845	103046214	-	-	0.021	-0.142	0.00006
rs2310300	103049074	-	-	0.022	-0.142	0.00006

rs10166330	103050390	-	-	0.009	-0.088	0.06110
rs3755265	103052816	-	0.052	0.018	-0.142	0.00006
rs10176820	103054420	-	-	0.009	-0.089	0.05920
rs2058659	103054556	-	0.053	0.016	-0.142	0.00006
rs13021177	103056493	-	0.042	0.029	-0.143	0.00005
rs2075186	103057251	-	-	0.002	-0.091	0.05410
rs11465736	103067930	-	-	2.79e-05	-0.096	0.04130
rs17821875	103071030	-	-	0.127	-0.014	0.82500
rs10169676	103074919	-	-	2.87e-05	-0.089	0.05970
rs10210176	103079516	-	-	0.001	-0.082	0.08120
rs10172116	103087573	-	-	2.31e-04	-0.110	0.02050
rs1403552	103088777	-	-	0.001	-0.088	0.06140
rs7597819	103092906	-	-	0.021	0.113	0.00947
rs6737668	103093081	-	0.026	0.027	0.113	0.00941
rs10469840	103093243	-	-	0.032	0.107	0.01430
rs759382	103094213	-	0.011	-	0.105	0.01420
rs9989749	103123642	-	0.033	-	0.065	0.15300
rs6751949	103125138	-	0.009	-	0.070	0.11200
rs4851607	103125632	-	0.002	-	0.079	0.07400
rs10195948	103125736	-	0.004	-	0.085	0.05510
rs2192757	103132378	-	0.004	-	0.080	0.06250
rs1916307	103134180	-0.042	-	-	0.089	0.03680
rs1523203	103135759	-	0.020	-	0.082	0.05570
rs1403551	103136446	-	0.021	-	0.082	0.05510
rs4851613	103137990	-	0.021	-	0.084	0.05080
rs6750851	103138761	-	0.021	-	0.081	0.05790
rs6750971	103138825	-	0.021	-	0.083	0.05240
rs10193407	103139298	-	0.042	-	0.083	0.05350
rs17774619	103216776	0.115	-	-	-0.393	0.02020
rs1016160	103245025	-	-	0.011	0.086	0.09020
rs17027893	103370379	-	-0.008	-	-0.022	0.65500
rs10172680	103376383	-	-0.011	-	-0.011	0.81800
rs2080294	103382425	-	-0.012	-	-0.012	0.81600
rs6705433	103386225	-	-0.011	-	-0.016	0.74600
rs6705568	103386368	-	-0.010	-	-0.014	0.77400
rs2310358	103413658	-	-0.041	-	-0.076	0.27400
rs75535834	103784955	0.154	-	-	0.157	0.29300
rs6740972	103837781	0.028	-	-	-0.042	0.29600
rs7582789	103860075	-0.003	-	-	0.126	0.20300

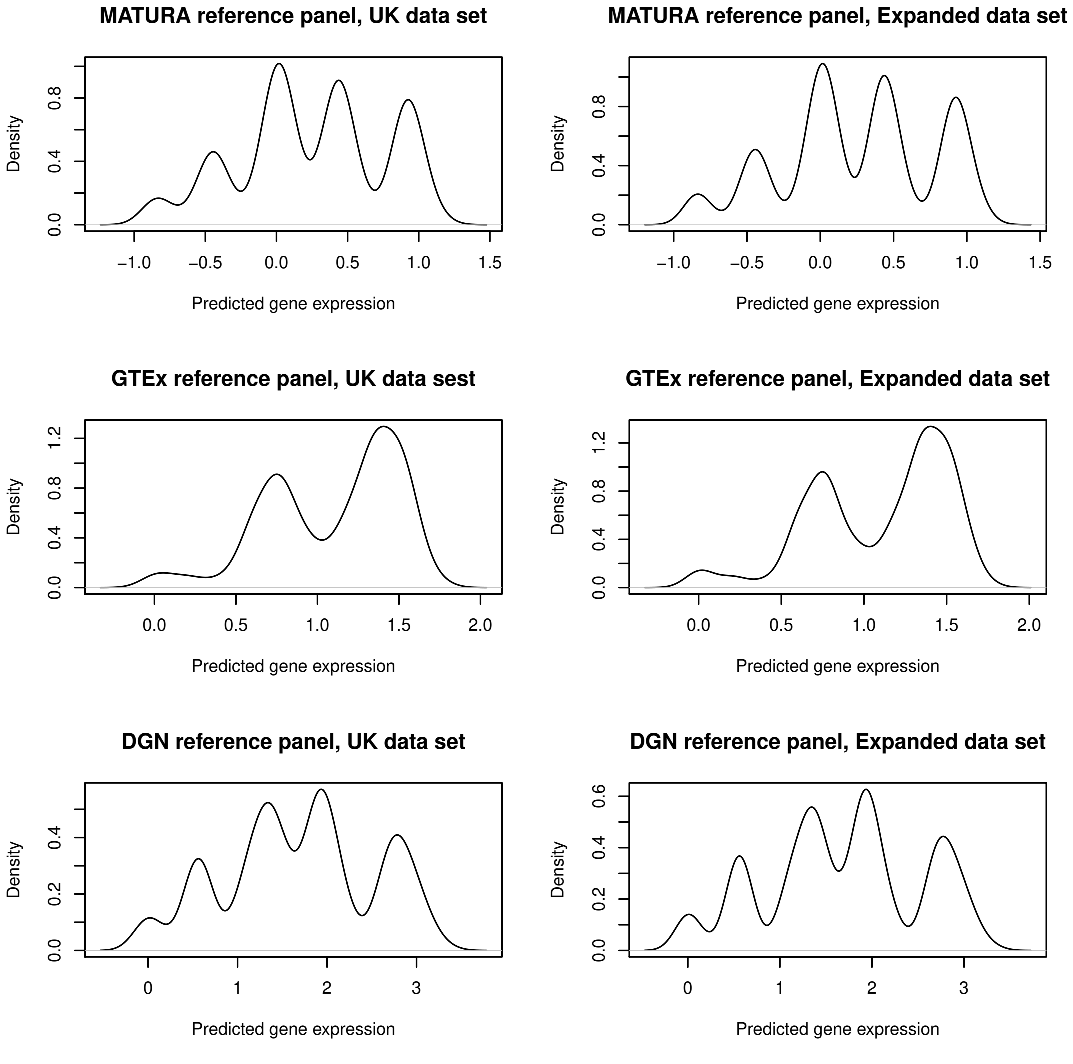

Figure S1. Density estimates (calculated using the density() function in R) of the predicted genetically-regulated gene expression at *IL18RAP* in the UK and expanded European ancestry cohorts.

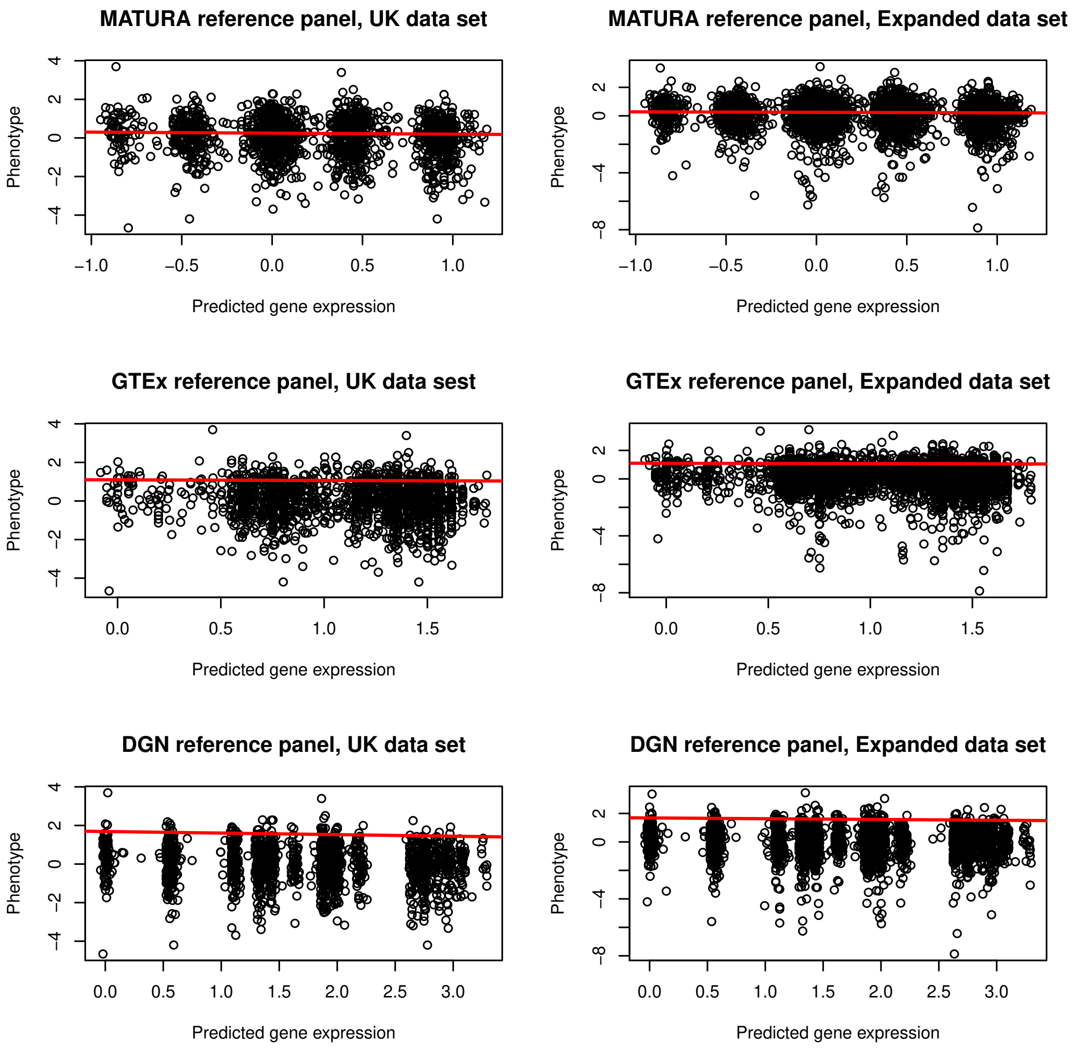

Figure S2. Plots of the linear regressions that are being fitted at *IL18RAP* in the UK and expanded European ancestry cohorts. Estimated regression lines are shown in red.
